# Hygienic Behavior of Africanized Honey Bees *Apis mellifera* Directed towards Brood in Old and New Combs during Diurnal and Nocturnal Periods

**DOI:** 10.3390/insects4040521

**Published:** 2013-09-26

**Authors:** Rogério A. Pereira, Michelle M. Morais, Tiago M. Francoy, Lionel S. Gonçalves

**Affiliations:** 1 Department of Genetics, Faculty of Medicine of Ribeirão Preto, São Paulo University, Ribeirão Preto, SP 14040-901, Brazil; E-Mail: lsgoncal@usp.br; 2 Science and Technology Department, Federal University of São Paulo, São José dos Campos, SP 12231-280, Brazil; E-Mail: michelle.manfrini@unifesp.br; 3 School of Arts, Sciences and Humanities, São Paulo University, São Paulo, SP 03828-000, Brazil; E-Mail: tfrancoy@usp.br; 4 Department of Animal Sciences, Federal Rural University of Semiarid, Mossoró, RN 59625-900, Brazil

**Keywords:** hygienic behavior, *Apis mellifera*, social insects, behavior of bees, Africanized honey bees

## Abstract

Hygienic behavior in honey bees, *Apis mellifera*, is measured by determining the rate at which the bees uncap and remove dead sealed brood. We analyzed individual behavior of house-cleaning Africanized honey bees in order to focus on some poorly understood aspects of hygienic behavior. Two observation hives, each with approximately 3,000 individually marked bees, were used in this study. The efficiency of hygienic behavior was evaluated in hygienic and non-hygienic strains of bees using two types of combs (new and old), as well as at different periods of the day (night and day). We also recorded the age of workers that performed this task of removing dead brood. In both strains, the workers that performed tasks related to hygienic behavior were within the same age cohort; we found no influence of age on the amount of time dedicated to the task, independent of the type of comb or period of the day. The total time from perforation of the cell capping until the dead brood had been completely removed, and was significantly shorter during daytime than at night. Hygienic behavior directed towards dead brood in new combs was also significantly more efficient (faster) than for brood in old combs. The type of comb had significantly more effect than did the time of day. We conclude that the type of comb and time of day should be taken into consideration when evaluating hygienic behavior in honey bees.

## 1. Introduction

Behavioral studies of social insects, such as ants and honey bees, have revealed an elaborate system of division of labor [1]. Division of labor in honey bees is one of the most highly studied phenomena involving animal behavior [2]. Workers bees develop a series of tasks during their adult life, starting with tasks performed within the nest and going on at later ages to tasks outside the nest [3].

Division of labor in honey bee colonies is an extremely important attribute that contributes to their ecological success, though there is a considerable inter-individual variability [3]. Even among workers of similar age, task specialization can be seen [4]. Nevertheless, many behaviors, including grooming [5], guarding [6], foraging for pollen, nectar or water [7] and cleaning the nest [8,9] tend to be performed by bees of specific age ranges. Bees have also been observed removing sick or dead brood, which has been named “hygienic behavior”. Hygienic behavior of a bee is defined as the ability to detect and uncap cells with dead or diseased brood and remove it from the nest [10]. It is considered a major mechanism of resistance against parasites and pathogens [11,12] and has been the focus of numerous studies [13,14,15,16]. This behavior plays a key role in social immunity in honey bee colonies [17], since removing sick or dead bees helps maintain colony health [18].

It is known that hygienic behavior has a genetic basis. Early studies proposed a model with two loci [8,9] in the inheritance of this behavior in honeybees, indicating that it is controlled by two pairs of recessive genes, which in homozygous state cause bees to be hygienic. These bees are prone to detect and uncap affected brood cells and remove dead or diseased brood from the comb.

The number of genes involved in hygienic behavior is still not well elucidated; however, most studies have concluded that this behavior is controlled by two or more loci [8,9,19,20,21,22,23,24,25,26,27].

Although hygienic behavior is one of the best-studied areas in honey bee research, there are still relevant unanswered questions. These include the influence of extrinsic factors, such as the colony conditions, food availability [28], and type of comb [29]. We designed our study to determine whether type of comb, bee age and time of day affect hygienic behavior efficiency.

We decided to make a daily analysis of this behavior, because most research that has examined hygienic behavior in bees have made only periodic observations [24,30,31,32], which could mask details of this behavior.

## 2. Experimental Section

### 2.1. Selection of Colonies to Supply Bees

For the choice of bee colonies to be included in the study, initial tests for hygienic behavior were performed using the pin-killing test [33] in 70 colonies of Africanized honey bees kept in the experimental apiary of the Department of Genetics, Faculty of Medicine of Ribeirão Preto of the University of São Paulo. The colonies were then classified into three different groups according to the percentage of brood removed 24 h after pin-killing the brood. The colonies that removed an average of 80% or more of the dead brood were classified as hygienic and colonies with an average of 30% or less, as non-hygienic colonies. Colonies that removed between 31% and 79% were classified as intermediate and were not used in this study. The percentages were obtained from three consecutive tests conducted 15–18 days apart. Three hygienic colonies and three non-hygienic colonies were selected and served as donors to provide newly emerged bees that were marked and introduced into the observation hives.

### 2.2. Observation Hives

The observation hives consisted of a wooden frame measuring 53 cm × 53 cm × 34 cm, built according to the Langstroth hive model. The side walls of the hive consisted of removable glass sheets, 3 mm thick. In the front opening of the hive, a transparent polyethylene tube was inserted into a hole made in the wall of the observation room, giving the bees free access to the outside. Initially, each observation colony consisted of a frame of brood containing unmarked adult worker bees of various ages (to maintain the normal age structure of workers), food (pollen and honey) and a laying mated queen. To ensure a majority of marked bees within the colony, the brood frames were changed every 15 days for other frames containing only eggs and younger larvae to avoid the emergence of (unmarked) offspring.

### 2.3. Marking Bees

After the priori selection of colonies in the experimental apiary, frames containing brood about to emerge were collected and transferred to wooden cages measuring 46 cm × 25 cm × 7 cm, with both sides covered by a metal screen and maintained inside an incubator with temperature and humidity controlled at around 34 °C and 70%, respectively. Each day, 400 newly emerged workers (200 hygienic and 200 non-hygienic) were marked with numbered and colored labels glued to the thorax, for individual identification of each bee. The color and number code that we used allowed individual identification of more than 4,000 bees.

### 2.4. Filming Behaviors

Two video cameras attached to color TV monitors (one for the hygienic colony and another for the non-hygienic colony) were used to study the activities involved in hygienic behavior. To avoid disturbance of the colonies during filming, they were covered with red cellophane, leaving only the area that was being filmed exposed to a cool white light, provided by a fiber optic lighting system.

### 2.5. Provision of New and Old Combs to Study Hygienic Behavior

**Figure 1:**
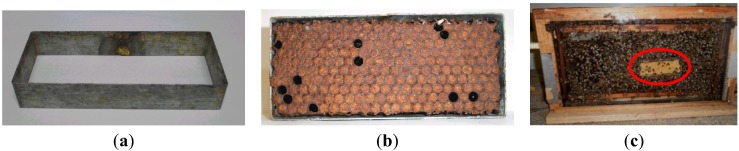
Comb insert used for filming individual behaviors. (**a**) metal support; (**b**) piece of worker brood comb inserted into the metal support; (**c**) test comb inserted into a brood frame (circled area) maintained in the observation hive.

A metal support frame, 12 cm × 5 cm (Figure 1a), was used to insert a piece of comb containing capped brood (Figure 1b), which was introduced into the observation hive (Figure 1c). The new combs were obtained from recently captured swarms, and the old combs were obtained from established colonies maintained in the apiary.

After insertion of comb inserts into the brood combs, these were placed in the observation hives to initiate the experiment. The brood (bee pupae) was killed by the perforation method (pin-killing). For daily observations, an area containing 40 capped brood cells was chosen; 20 cells were perforated and 20 served as controls. After perforation of the brood cells, they were filmed for 24 h, or until all of the perforated brood had been removed.

To ensure an unobstructed view of the bees that were working on the dead brood, the comb insert assembly was inserted only partially into the brood comb. This permitted only one layer of bees within the space between the glass wall of the observation colony and the comb insert, so that a second layer of bees could not obstruct the observations.

### 2.6. Hygienic Behavior at Different Times of Day

To determine whether there were differences in hygienic behavior during the day, the combs were perforated at different times. For the assessment of the behavior during daytime, the brood cells were perforated between 8:00 and 9:00 and for the night-time observations, they were perforated between 17:30 and 18:30.

### 2.7. Statistical Analysis

By reviewing the videos, we calculated the time spent, in seconds, by each bee that performed the different tasks that comprise hygienic behavior. Since the data had three different types of interaction (hygienic or non-hygienic strain, type of comb and period of the day), it was analyzed using a three-way ANOVA. The time spent uncapping and removing dead brood by individual workers was tested for normality with Shapiro-Wilk’s Normality test; after that it was tested for variance homogeneity using the Bartlett test of homogeneity of variances. Since the variances were not homogeneous, the data was transformed using a Box Cox transformation; after that, it was analyzed using a three-way ANOVA. When the three-way ANOVA showed differences, the differences between pairs were examined using the Tukey test (α = 0.05).

## 3. Results and Discussion

**Figure 2:**
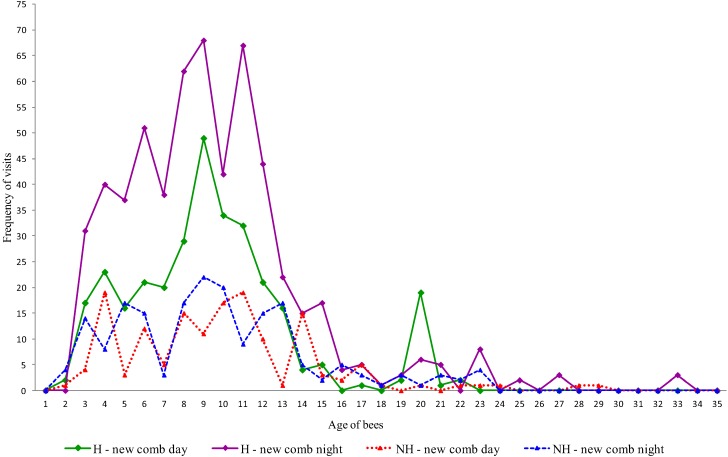
Age (in days) of workers that performed hygienic behavior on new combs in hygienic (H) and non-hygienic (NH) colonies at different times of day.

**Figure 3:**
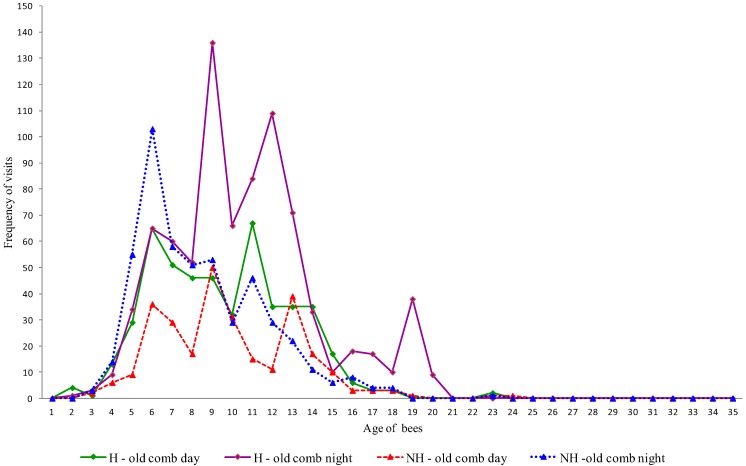
Age (in days) of workers that performed hygienic behavior on old combs in hygienic (H) and non-hygienic (NH) colonies at different times of day.

We recorded 7,303 h of the bee behaviors, of which 3,694 h were observations of hygienic colonies and 3,609 h of non-hygienic colonies. We found that the workers that performed tasks related to hygienic behavior (uncapping the cells and removing dead brood) were from the same age cohort, in both hygienic and non-hygienic strains of bees, independent of the period of the day or type of comb (Figure 2 and Figure 3). The youngest workers that displayed this behavior in both strains were two days old; however, the frequency at this age was low. The greatest concentration of bees performing hygienic behavior on new combs involved those that were 4–12 days of age (Figure 2), while on the old combs, the age range of maximum activity was between 6 and 13 days old (Figure 3).

Previous studies reported that bees performing hygienic behavior were mostly from 6–21 days old [30,32,34,35,36,37,38]. Recent studies have also observed bees less than five days old uncapping cells, with some observations involving bees only one day old [38]. Differences between reports in relation to the age of the workers that perform hygienic behavior may be related to genotype, since the age of bees that perform tasks can vary significantly from colony to colony [31]. Research involving integration of circadian rhythms and division of labor suggests that these differences are correlated with genotypic variation in the rate of behavioral development; genotypes of bees that progress through the age polyethism schedule faster also acquire behavioral rhythmicity at an earlier age. Although some bees are initially arrhythmic with respect to task performance, they develop circadian rhythmicity by reducing activity at night prior to becoming foragers [39].

When we analyzed the time spent on activities related to uncapping cells, a three-way ANOVA showed that the individual factors type of comb and period of the day were associated with significant (*p* < 0.0001) differences in hygienic behavior. Among these factors, significant interactions were found only between hygienic strains and period of the day (*p* < 0.001). All the other interactions were not significant at α = 0.05.

When we analyzed the interaction between hygienic behavior and the period of the day, the Tukey Test showed that at a confidence level of 95%, there were significant differences between periods of the day within the hygienic lines. This means that, although individual workers from the hygienic strains spent less time uncapping the cells during the day than they did during the night, the total time spent on this task was much greater at night (46,248 s) than during the day (12,332 s). This is because of the much greater number of visits during the nocturnal periods and the longer duration of this behavior at night (Table 1). On the other hand, although the same is not true for the non-hygienic strain regarding individual bees (Table 1), the time spent performing the tasks had the same tendency (30,890 s during the night × 9,152 s during the day). Since there were no significant differences in the time spent by bees from hygienic and non-hygienic strains in the same period of the day, this difference is most likely related to the number of bees performing uncapping tasks for hygienic behavior (Table 1). The number of bees that uncap cells at night is higher than during the day, possibly because workers are also performing other tasks inside the nest during the day, such as nectar reception and dehydration. This is logical since bees performing these tasks are of a similar age. However, without the food flow during the night, a greater number of workers can spend more time on hygienic behavior almost tripling the time spent uncapping the cells making this behavior more efficient during this period. Bees of the hygienic strain spent significantly more time uncapping cells from old combs than cells from new combs (*p* < 0.001). The influence of the type of comb is probably due to the existence of substances deposited on the wax of old combs, such as propolis and water soluble substances. These substances contribute to the increased thickness and cell wall resistance of the old combs. On the other hand, a new comb is nearly pure beeswax, with thin and more fragile cell walls, which could facilitate uncapping [40]. However, although bees spent more time uncapping cells from old combs, the final result observed in the total removal of dead brood was similar for new and old combs. This is probably due to the number of visits bees made to old and new combs, leading to a similar uncapping rate in both types of combs. 

**Table 1:** Median and quartiles of time spent by workers of hygienic (H) and non-hygienic (NH) strains to uncap perforated cells of old and new brood combs during different times of day. Different letters in the same column indicate significant differences. Same letters in the same column indicate absence of significant differences (α = 0.05).

UNCAPPING				
Strain/type of comb/period	N	Median	25%	75%
H/new/day	177	19.0 ^a^	11.0	29.0
H/new/night	327	16.0 ^b^	11.0	30.7
H/old/day	333	38.0 ^c^	14.0	61.5
H/old/night	760	22.0 ^c^	10.0	57.0
NH/new/day	151	17.0 ª	11.0	35.5
NH/new/night	151	20.0 ª	12.0	32.0
NH/old/day	258	29.0 ^c^	14.0	48.0
NH/old/night	501	25.0 ^c^	14.0	43.0

When we examined the time spent to remove dead brood, the three-way ANOVA indicated no significant differences (*p* = 0.23) between the periods of the day. It can therefore be assumed that this factor does not influence this task. However, there were significant interactions between the different strains (*p* < 0.001) and also between the types of comb (*p* < 0.0001). All the other interactions were not significant at a confidence level of 95% (Table 2).

**Table 2:** Median and quartiles of time spent by workers of hygienic (H) and non-hygienic (NH) strains to remove dead brood from old and new brood combs at different periods of the day. Different letters in the same column indicate significant differences. Same letters in the same column indicate absence of significant differences (α = 0.05).

REMOVAL				
Strain/type of comb/period	N	Median	25%	75%
H/new/day	339	390 ª	229	657
H/new/night	863	388 ª	210	644
H/old/day	395	311 ^b^	185	464
H/old/night	655	316 ^b^	179	533
NH/new/day	153	477 ^c^	253	808
NH/new/night	201	556 ^c^	269	811
NH/old/day	123	301 ^b^	179	448
NH/old/night	183	337 ^b^	190	475

When we analyzed interactions between strains, we found that although individual bees from the hygienic strain spent significantly less time removing dead pupae than bees from the non-hygienic strain (*p* = 0.003), the overall time spent on this task was approximately three times greater in the hygienic colonies (H 949,148 s × NH 313,765 s. Allied to the fact that colonies from the hygienic line spent more time removing the dead pupae, they also tended to do more removals without cannibalizing the pupae (total removal), which made the process more efficient (faster) when compared to the removal of dead pupae in the non-hygienic colonies [40].

**Figure 4:**
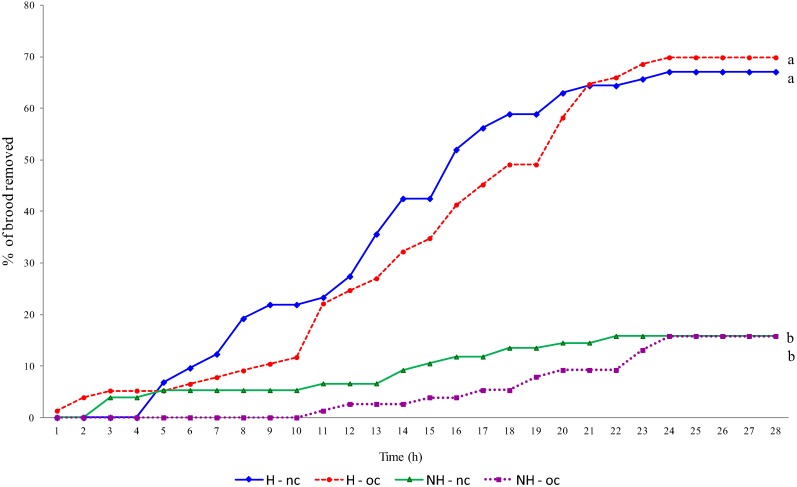
Percentage of dead brood removed hour by hour in hygienic and non-hygienic colonies after perforation of the brood. Same letters at the end of each line indicate absence of significant differences. Different letters at the end of each line indicate significant differences (Mann-Whitney Rank Sum Test, *p* < 0.001).

When we examined the interactions between types of comb and genetic strains, bees working on new combs generally spent more time performing the removal tasks than bees doing the same task on old combs (*p* = 0.001). Joining of the two interactions demonstrated that the same pattern was repeatedly found; bees from hygienic and non-hygienic colonies spent more time removing dead brood from new combs than from old combs (*p* = 0.001). We also found that the efficiency of this behavior was greater in the new combs for both hygienic and non-hygienic lines, reaching greater levels of removal in less time. However, the final result was practically the same after 24 h (Figure 4).

Additionally, we compared the total time spent by workers from the introduction of the perforated cells until total removal of dead brood. The hygienic bees were significantly faster at detecting and removing dead brood from the cells than the non-hygienic bees (*p* < 0.001; Figure 4). In some cases, in the hygienic colony, the hygienic behavior was so efficient that at one hour after perforation of the cells, all of the brood had been removed.

Our results support conclusions from previous reports, demonstrating the efficiency of workers in the rapid detection and removal of dead brood from the cells [41]. One hour after perforation of the brood cells, hygienic bees had removed 43%, while the non-hygienic bees removed only 25%. Hygienic bees finished the removal in 15 h, whereas the non-hygienic bees took over 30 h.

Significant differences in the uncapping of the cells were found in the comparisons involving both the type of comb and the time of day when the activities occurred. We can infer that together with the genetic factor, the use of new combs can be important when studying the behavior of individual workers as they perform hygienic behavior. However, the differences related to the type of comb were found only at the individual bee level. The final results found in the completion of the hygienic behavior by these bees was practically the same (Figure 4) and possibly the type of comb has no biologically important influence.

Although new combs influenced individual hygienic behavior, we reaffirm that the major factor influencing this behavior is genetic. Here, we have provided new evidence about differences in brood cell cleaning behavior in hygienic and non-hygienic colonies. In most cases (except for removal time), the time spent by individual bees from the two strains was very similar, and the differences in the final results were due mainly to the number of bees performing the tasks, which results in significant differences in the amount of time spent by the colonies. These factors may prove to be important to beekeepers who want to select bees for hygienic behavior to increase disease resistance rather than treating them with antibiotics, fungicides, or through other means.

## 4. Conclusions

Here, we shed light on how hygienic behavior is performed by honey bees in hygienic and non-hygienic colonies on different types of combs. We conclude that in both strains, and on both types of combs, hygienic behavior is performed similarly in the various age cohorts, with young ages being more actively involved. Furthermore, we concluded that the bees work with the same efficiency in both types of combs, independent of age. Individual bees spend more time uncapping the cells in hygienic colonies during the day than at night; however, the total time is much greater during the night than during the day. This is because more bees are involved at night. We also confirmed that bees in hygienic colonies are more efficient in detection and removal of dead brood than workers of non-hygienic colonies. We concluded finally, that there is a tendency for hygienic behavior to be more efficient in new combs than in old combs in the initial contact with the killed brood; but the final results after 24 h are similar. We conclude that genetic tendencies are the main determinants for efficiency of hygienic behavior; however, the type of comb and time of day are also important factors that should be taken into consideration when studying individual behavior of bees performing hygienic behavior. These findings are important for beekeeping due to the importance of selecting colonies for high hygienic behavior tendency.
